# qInward variability-based *in-silico* proarrhythmic risk assessment of drugs using deep learning model

**DOI:** 10.3389/fphys.2022.1080190

**Published:** 2022-12-14

**Authors:** Da Un Jeong, Nurul Qashri Mahardika T, Aroli Marcellinus, Ki Moo Lim

**Affiliations:** ^1^ Department of IT Convergence Engineering, Kumoh National Institute of Technology, Gumi, Gyeongbuk, South Korea; ^2^ Department of Medical IT Convergence Engineering, Kumoh National Institute of Technology, Gumi, Gyeongbuk, South Korea; ^3^ Meta Heart Inc., Gumi, Gyeongbuk, South Korea

**Keywords:** torsades de pointes (TdP), proarrhythmic risk, drug toxicity, qInward variability, convolutional neural network (CNN)

## Abstract

Many researchers have suggested evaluation methods and Torsades de Pointes (TdP) metrics to assess the proarrhythmic risk of a drug based on the *in silico* simulation, as part of the Comprehensive *in-vitro* Proarrhythmia Assay (CiPA) project. In the previous study, we validated the robustness of 12 *in silico* features using the ordinal logistic regression (OLR) model by comparing the classification performances of metrics according to the *in-vitro* experimental datasets used; however, the OLR model using 12 *in silico* features did not provide desirable results. This study proposed a convolutional neural network (CNN) model using the variability of promising *in silico* TdP metrics hypothesizing that the variability of *in silico* features based on beats has more information than the single value of *in silico* features. We performed the action potential (AP) simulation using a human ventricular myocyte model to calculate seven *in silico* features representing the electrophysiological cell states of drug effects over 1,000 beats: qNet, qInward, intracellular calcium duration at returning to 50% baseline (CaD50) and 90% baseline (CaD90), AP duration at 50% repolarization (APD50) and 90% repolarization (APD90), and dVm/dtMax_repol. The proposed CNN classifier was trained using 12 train drugs and tested using 16 test drugs among CiPA drugs. The torsadogenic risk of drugs was classified as high, intermediate, and low risks. We determined the CNN classifier by comparing the classification performance according to the variabilities of seven *in silico* biomarkers computed from the *in silico* drug simulation using the Chantest dataset. The proposed CNN classifier performed the best when using qInward variability to classify the TdP-risk drugs with 0.94 AUC for high risk and 0.93 AUC for low risk. In addition, the final CNN classifier was validated using the qInward variability obtained after merging three *in-vitro* datasets, but the model performance decreased to a moderate level of 0.75 and 0.78 AUC. These results suggest the need for the proposed CNN model to be trained and tested using various types of drugs.

## 1 Introduction

Torsades de Pointes (TdP) is a fatal arrhythmia induced by drugs. The major challenge in developing new drugs is to filter life-threatening drugs, which can lead to TdP, and prevent them from entering the market (Sager et al., 2014; Vicente et al., 2018). From the 1990s to the early 2000s, many drugs were withdrawn from the market due to concerns about inducing TdP (Roden, 2004; Onakpoya et al., 2016; Vicente et al., 2018). To detect drugs that have the risk of TdP, the International Council on Harmonization (ICH) has established a guideline based on the mechanism of pharmaceutically occurring QT interval prolongation, which is blockage of the I_Kr_ channel, a human ether-à-go-go-related gene (hERG) channel current (International Council on Harmonisation, 2005a; Harmonisation, 2005b). Although this guideline can accurately detect proarrhythmic drugs in new drug development, the strict regulation due to the low specificity of this guideline may disrupt the development of a potential therapeutic drug that prolongs the QT interval but never leads to TdP (Llopis-Lorente et al., 2020).

To address this problem, a comprehensive proarrhythmia assay (CiPA) was proposed at a Think Tank meeting at the US Food and Drug Administration (FDA) headquarters in 2013 (Sager et al., 2014; Strauss et al., 2019). CiPA, as a new paradigm, assesses the proarrhythmogenic risk of a drug through *in silico* simulation using a human cardiomyocyte model integrating multi-ion channels of pharmacological *in-vitro* data (Li et al., 2019). As part of this CiPA project, many researchers have suggested evaluation methods and TdP metrics to assess the proarrhythmic risk of drugs based on the *in silico* simulation. Lancaster and Sobie (2016) suggested several metrics derived from action potential (AP) and intracellular calcium (Ca^2+^) concentration from *in silico* simulations observing drug response in human ventricular cell models, such as AP duration at 50% repolarization (APD_50_), AP duration at 90% repolarization (APD_90_), Ca duration at getting to 50% baseline (CaD_50_), and Ca duration at getting to 90% baseline (CaD_90_). The FDA proposed qInward and qNet as promising TdP risk metrics computed from *in silico* simulations for categorizing the proarrhythmic risk of drugs as high, intermediate, and low (Chang et al., 2017a; Li et al., 2017, 2019). Through several studies, some of these *in silico* features were reported to be highly accurate in classifying the torsadogenic and non-torsadogenic drugs.

In our previous study, we validated the robustness of 12 *in silico* features using an ordinal logistic regression (OLR) model by comparing the classification performances of metrics according to the used *in-vitro* experimental datasets (Jeong et al., 2022a). However, as the results of the OLR model using 12 *in silico* features were desirable, the single value of the *in silico* feature was perceived as limiting to classifying the three TdP risks. Therefore, this study proposes a convolutional neural network (CNN) model using the variability of promising *in silico* TdP metrics hypothesizing that the variability of *in silico* features based on beats contains more information than the single value of *in silico* features. First, we determined a CNN classifier by comparing the classification performance of the proposed model according to the variabilities of seven *in silico* biomarkers obtained from *in silico* drug simulation using specific *in-vitro* data. We then evaluated the robustness of the proposed CNN classifier using the variability of *in silico* biomarkers obtained from different *in-vitro* data.

## 2 Methods

### 2.1 In-vitro experimental dataset and preprocessing

In this study, to determine TdP-risk, an *in-vitro* experimental dataset for CiPA 28 drugs measured by Chantest et al. of Charles Rivers Laboratories (Han et al., 2020) was used to train and test the proposed CNN classifier. The finalized model tested using the Chantest datasets was validated using two *in-vitro* experimental datasets of Li et al. (2019) and Nanion et al. (Han et al., 2020) (Supplementary Table S1). These three datasets include the inhibition rates of seven ionic channels according to the concentration of the corresponding CiPA 28 drugs; the CiPA drugs were classified as high-risk, intermediate-risk, and low-risk according to their proarrhythmic risk. The uncertainty quantification algorithm was used for reliability and sufficient inputs by bootstrapping the experimental datasets based on the Markov-Chain Monte Carlo (MCMC) method (Chang et al., 2017b), which generated 2,000 Hill curves that demonstrated the relationship between ion channel block and drug concentration. The drug concentration causing half-maximal inhibition of the ionic channel (IC50) and the slope at IC50 (H, Hill coefficient) were obtained from these Hill curves and used as inputs for the *in silico* simulation. We used 2,000 samples of IC50 and H per drug for obtaining the *in silico* features reflecting the drug effect.

### 2.2 *In-silico* model

This study used the Tomek-Ohara Rudy model (ToR-ORD model), which was developed to integrate and delineate well-mimicked healthy and diseased hearts according to drug effects by (O’Hara et al., 2011; Dutta et al., 2017; Tomek et al., 2019). The ToR-ORD model has been revised using the I_CaL_, I_NaCa_, and I_Kr_ equations to reproduce the AP shape, transient calcium concentration, and sodium homeostasis under conditions reflecting the plateau potentials of experimental data. The drug effects were applied to myocytes using the following equation, consisting of IC50, H, and drug concentration (D):

$$Inhibitionfactor=\frac{1}{1+{\left(IC50/\left[D\right]\right)}^{H}}$$ (1)

**FIGURE 1:**
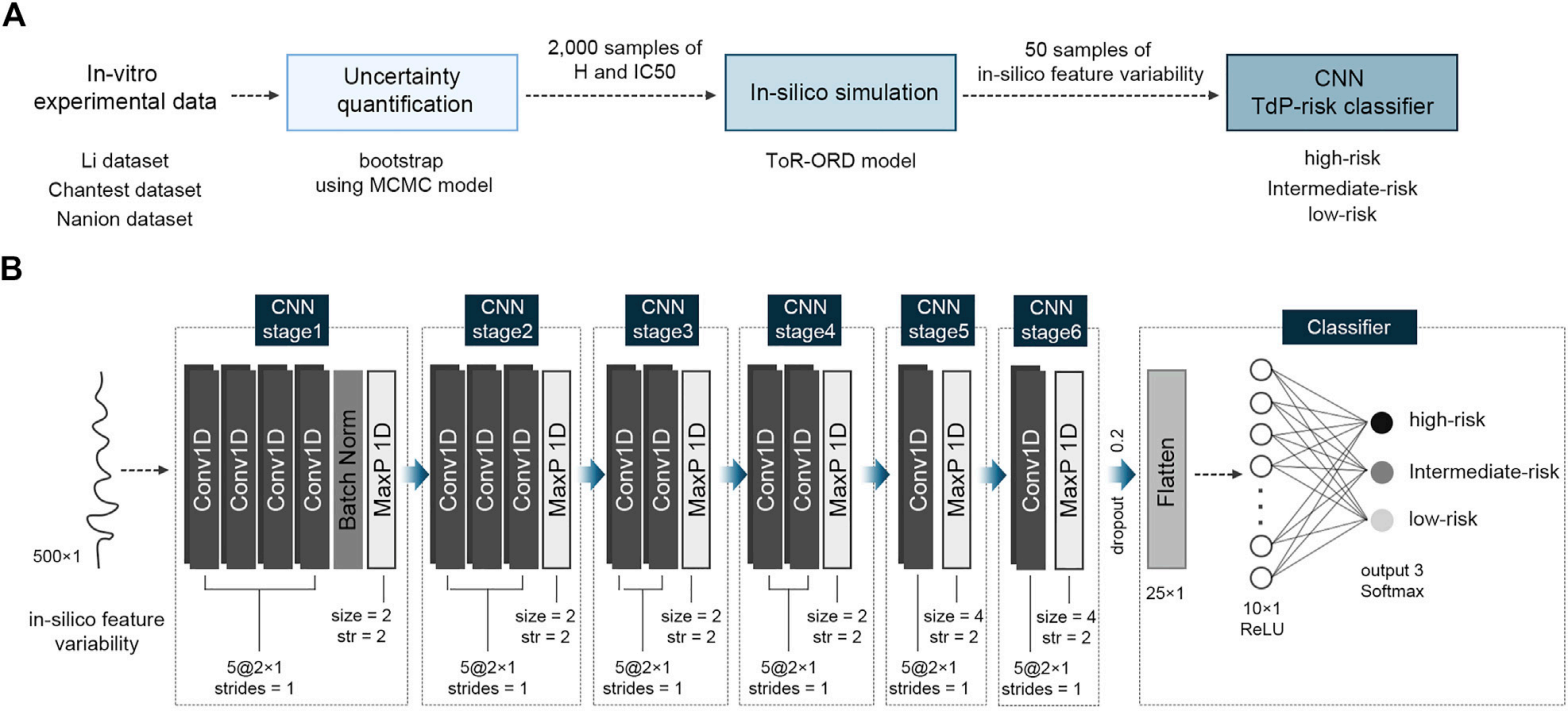
Schematic of the proposed algorithm for TdP-risk assessment; **(A)**, flow chart of the process; **(B)**, the proposed convolutional neural network (CNN) classifier using *in silico* feature variability; MCMC, Markov-chain Monte Carlo; H, Hill coefficients; IC50, the half inhibitory concentration; Conv1D, one-dimensional CNN layer; Batch Norm, Batch Normalization; MaxP 1D, one-dimensional max pooling layer; str, strides; ReLU, Rectified Linear Unit activation function.

In-silico simulation was performed according to the four changes of 1×, 2×, 3×, and 4× in maximum plasma concentration of the drug (Cmax) (Table1). Therefore, for each Cmax variation, there were 2,000 samples of *in silico* feature variability and thus, there were 8,000 samples per drug. Through simulations, the electrophysiological status of human ventricular myocytes was mathematically computed according to the drug effect during 1,000 pacings with 2,000 m of a cycle length (30 bpm of heart rate), which referred to the research of the FDA. Through drug simulation, we calculated seven *in silico* features representing the electrophysiological cell states for drug effects at every pacing: qNet, qInward, CaD_50_, CaD_90_, APD_50_, APD_90_, and dVm/dt_Max_repol_. qNet is the amount of ionic charge crossing the six ionic channels: I_NaL_, I_CaL_, I_Kr_, I_Ks_, I_K1_, and I_to_ Eq. 2; Chang et al., 2017b. qInward is a change in the amount of ionic charge moving inward through I_CaL_ and I_NaL_ (Eq. 3) here, it is noted that the cqInward used by Li et al. (2017) is the same concept qInward in this study.

$$qNet=\overset{CL}{\underset{0}{\int }}\left(I_{NaL}+I_{CaL}+I_{Kr}+I_{Ks}+I_{K1}+I_{to}\right)dt$$ (2)

$$qInward=\frac{1}{2}\left(\frac{AUC_{I_{CaL-drug}}}{AUC_{I_{CaL-control}}}+\frac{AUC_{I_{NaL-drug}}}{AUC_{I_{NaL-control}}}\right)$$ (3)

where CL is the cycle length and AUC is “the area under the curve,” which is the area under the ionic channel trace. CaD_50_, CaD_90_, APD_50_, and APD_90_ represent the time duration from the AP upstroke to 50% and 90% repolarization in the transient calcium and AP traces, respectively. dVm/dt_Max_repol_ denotes the repolarization velocity as the slope of the AP repolarization. As transient *in silico* feature variability is observed at the beginning of pacings, we extracted the *in silico* feature variance according to the last 500 beats under the steady state and fed them to the proposed CNN classifier as inputs. The overall process performed in this study to assess the TdP-risk of drugs is shown schematically in Figure 1A.

**TABLE 1:** Proarrhythmic risk level of CiPA 28 drugs.

Proarrhythmic risk level	CiPA 28 drugs			
	Train drugs	Cmax (nM)	Test drugs	Cmax (nM)
High	Bepridil	33	Azimilide	70
	Dofetilide	2	Disopyramide	742
	Sotalol	1,439	Ibutilide	100
	Quinidine	3,237	Vandetanib	255.4
Intermediate	Cisapride	2.6	Astemizole	0.26
	Chlorpromazine	38	Clarithromycin	1,206
	Ondansetron	139	Clozapine	71
	Terfenadine	4	Domperidone	19
			Droperidol	6.33
			Pimozide	0.431
			Risperidone	1.81
Low	Diltiazem	122	Loratadine	0.45
	Mexiletine	4,129	Metoprolol	1,800
	Ranolazine	1,948.2	Nifedipine	7.7
	Verapamil	81	Nitrendipine	3.02
			Tamoxifen	21

### 2.3 Proposed CNN classifier

Figure 1B shows the structure of the proposed CNN classifier with six CNN stages. All the CNN stages were group sets comprising a series of CNN layers and one max pooling layer: four CNN layers for the first stage, three CNN layers for the second stage, two CNN layers for the third and fourth stages, and one CNN layer for the fifth and sixth stages. Each CNN layer had five filters of 2 × 1 with one stride (moving one space), implying that every filter was applied by overlapping one data point. Through the max pooling layer in the first to fourth CNN stages, the maximum value in every 2 × 1 window was extracted with two strides, while in the fifth and sixth CNN stages, the max pooling size was 4 with every two strides (moving two spaces). To prevent overfitting of the proposed CNN model, a batch normalization layer was added at the end of the first CNN stage. After passing through the six CNN stages, two-dimensional machine learning features of 5 × 5 were flattened to 25 and fed into the hidden layer with ten neurons while randomly dropping out by 20%. Finally, the output layer classified the TdP-risk of a drug into three levels: high-, intermediate-, and low-risk. Apart from the output layer, the activation functions of all CNN layers and hidden layers were a “Rectified Linear Unit (ReLU)” function, and the output layer used a “Softmax” function.

The CNN classifier was trained using 12 drugs comprising three TdP-risk groups in equal proportions: four high-risk drugs, four intermediate-risk drugs, and four low-risk drugs. We randomly extracted 50 samples per drug from 8,000 (2,000 samples × four concentrations per drug) *in silico* feature variability samples and used only 600 samples (50 samples × 12 training drugs) for training the model. The model parameters were updated every 20-batch data during 300 training epochs. We obtained the best-fit model with hyperparameters, which had the highest performance in both training and validation, through 10-fold cross-validation. The model optimization function was an adaptive momentum (Adam) with a learning rate of 0.00001, and the loss function was a categorical cross-entropy function for predicting three TdP risk levels.

**FIGURE 2:**
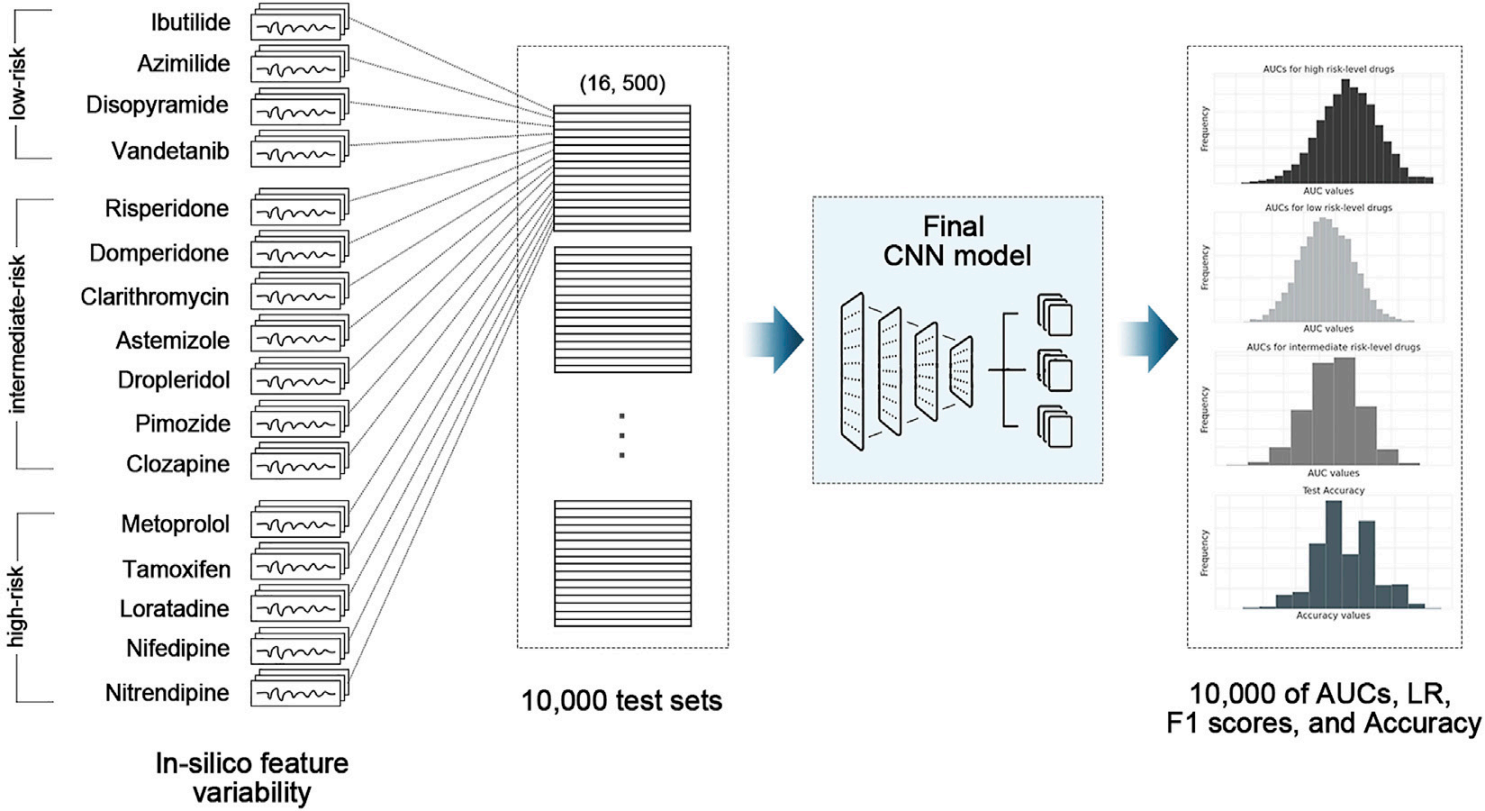
Schematic of 10,000-test algorithm; CNN, convolutional neural network model; AUC, area under the receiver operating curve; LR, likelihood ratio.

The final CNN model was tested using 16 drugs, comprising four high-risk drugs, seven intermediate-risk drugs, and five low-risk drugs, through the 10,000-test algorithms (Table 1); (Li et al., 2019). The test drug set consisted of 2,000 feature samples of *in silico* biomarkers for four concentrations per drug, a total of 128,000 samples (2,000 feature samples x four concentrations x 16 drugs). In the 10,000-test algorithms, we repeated the extraction of one sample (one instance per drug) among 8,000 (2,000 samples × four concentrations) *in silico* feature variabilities per drug, generating 10,000 test sets of 16 drugs. The final CNN classifier was tested 10,000 times, using 10,000 test sets, outputting 10,000 performance scores. We calculated the AUC of the receiver operating characteristic curve, likelihood ratio (LR), F1 score, and accuracy after 10,000 tests. We then compared the median, minimum, and maximum of the model performance indices according to the *in silico* feature variabilities Figure 2; (Jeong et al., 2022b).

## 3 Results

**FIGURE 3:**
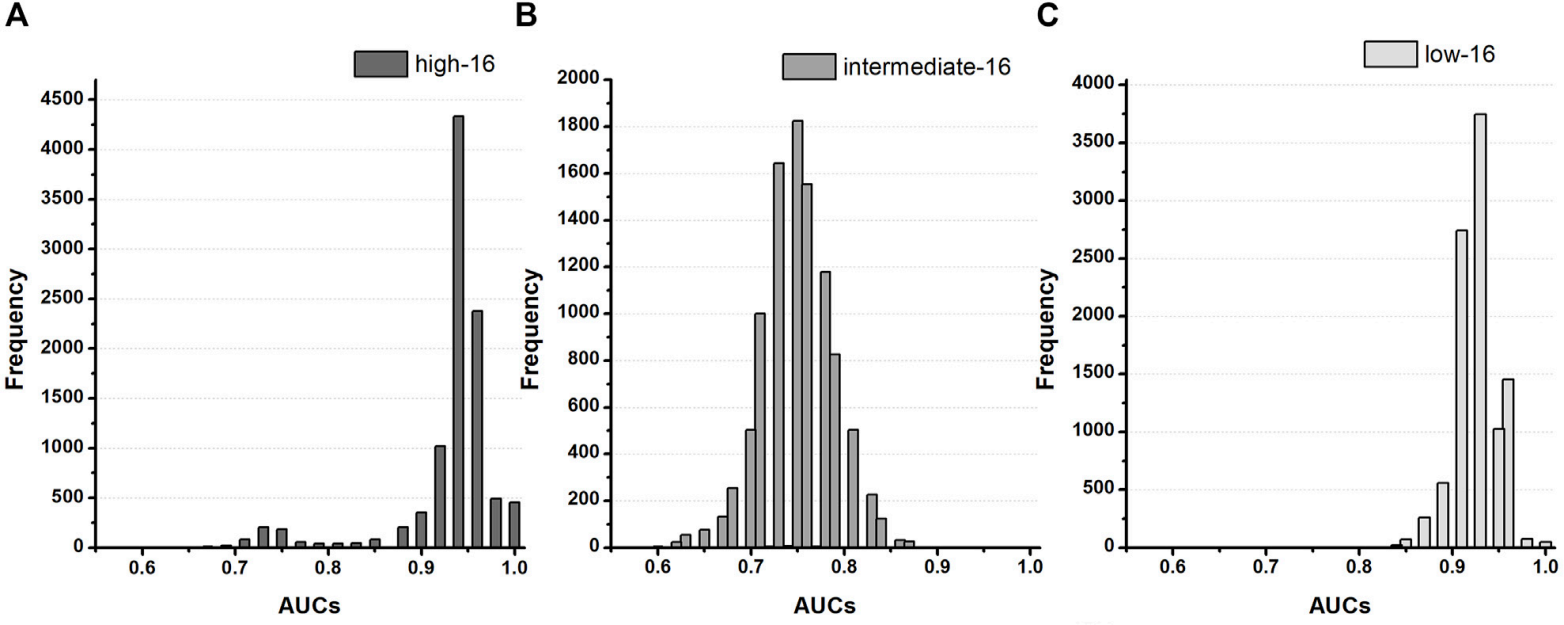
Distribution of AUCs based on the TdP-risk using qInward variability of 16 test drugs in the Chantest dataset; **(A–C)**, AUC distribution for the high, intermediate, and low-risk of the CNN classifier for 16 test drugs.

We demonstrated the performance of the proposed CNN classifier for 16 test drugs based on seven *in silico* feature variabilities calculated from drug simulation using *in-vitro* datasets of Chantest et al. (Table 2; Figure 3). The model performance was excellent for classifying high-risk and low-risk drugs with an AUC of 0.94 and 0.93, respectively, using qInward variability. Especially for the low-risk drugs, qInward variability demonstrated the best performance compared to other *in silico* feature variabilities, with less than or approximately 0.70 AUC. The classification accuracy of the intermediate-risk drugs using qInward variability with the CNN classifier was moderate at 0.75 AUC, but it was the highest among the *in silico* feature variabilities. In the CNN classifiers using qInward variability, the LR + and LR-for three TdP-risk were satisfied to over minimal limitation level the excellent level of LR + > 10 and LR− < 0.1, a suitable level for LR + > 5 and LR − < 0.2, and the minimally acceptable level for LR + > 2 and LR − < 0.5.

**TABLE 2:** CNN classifier performance for 16 test drugs according to the *in silico* feature variabilities; performance indexes represent the median, the minimum, and the maximum values as the results of 10,000 times test algorithms; Three asterisks (***) denote excellent performance over 0.9 of the median AUC value, two asterisks (**) for good performance over 0.8 of the median AUC value, and one asterisk (*) for moderate performance over 0.7 of the median AUC value.

Model		In-silico feature variability of chantest dataset						
		qNet	qInward	CaD_50_	CaD_90_	APD_50_	APD_90_	dVm/dt_max_repol_
AUC	High	0.83** (0.31–0.96)	0.94*** (0.60–1.00)	0.83** (0.54–1.00)	0.46 (0.19–0.90)	0.82** (0.63–0.94)	0.81** (0.67–0.92)	0.81** (0.56–0.94)
	Intermediate	0.62 (0.38–0.78)	0.75* (0.57–0.92)	0.71* (0.48–0.84)	0.71* (0.57–0.86)	0.70* (0.55–0.80)	0.73* (0.57–0.84)	0.72* (0.60–0.82)
	Low	0.64 (0.44–0.98)	0.93*** (0.82–1.00)	0.25 (0.01–0.51)	0.71* (0.23–0.65)	0.70* (0.49–0.88)	0.51 (0.36–0.89)	0.65 (0.46–0.78)
LR+	High	2.33 (0.56–Inf)	Inf (2.2–Inf)	0.00 (0.00–9.00)	0.50 (0.00–5.0)	3.00 (0.70–17.5)	1.44 (0.73–0.7)	3.00 (0.70–Inf)
	Intermediate	1.71 (0.96–6.00)	3.6 (1.04–Inf)	3.6 (1.04–6.00)	3.6 (0.96–7.71)	2.33 (0.83–4.12)	1.94 (0.80–4.67)	2.67 (1.04–10.0)
	Low	1.08 (0.55–4.67)	4.67 (2.89–6.5)	0.42 (0.00–1.08)	0.55 (0.42–2.5)	2.33 (0.00–3.75)	0.55 (0.00–2.89)	2.93 (0.58–4.8)
LR−	High	0.64 (0.00–1.19)	0.27 (0.00–0.73)	1.36 (0.00–1.50)	0.00 (0.00–1.40)	0.53 (0.00–1.14)	0.87 (0.00–1.1)	0.6 (0.16–1.14)
	Intermediate	0.64 (0.29–1.03)	0.48 (0.22–0.97)	0.48 (0.29–0.97)	0.48 (0.16–1.03)	0.56 (0.33–1.64)	0.62 (0.39–1.2)	0.55 (0.29–0.97)
	Low	0.96 (0.00–1.26)	0.00 (0.00–0.43)	1.39 (0.96–1.83)	1.26 (0.62–1.39)	0.75 (0.33–1.12)	1.26 (0.43–1.83)	0.43 (0.00–1.23)
Accuracy		0.50 (0.30–0.70)	0.74 (0.49–0.88)	0.45 (0.25–0.63)	0.53 (0.31–0.65)	0.54 (0.36–0.72)	0.35 (0.14–0.6)	0.58 (0.39–0.77)
F1 score		0.50 (0.31–0.69)	0.69 (0.50–0.88)	0.44 (0.25–0.56)	0.50 (0.31–0.62)	0.54 (0.36–0.94)	0.32 (0.12–0.62)	0.57 (0.39–0.75)

The proposed CNN classifiers using the *in silico* feature variabilities mostly classified the high-risk drugs with a good performance of over 0.80 AUCs, apart from CaD_90_ variabilities, which demonstrated very poor performance of 0.46 AUC. CaD_90_ variability showed only a medium level of accuracy for the other two groups of TdP-risk drugs (both 0.71 AUCs). The intermediate-risk drugs were classified at a moderate accuracy of approximately 0.70 AUCs through the proposed CNN classifier with six *in silico* feature variabilities, except for qNet variability. qNet variability classified only high-risk drugs effectively, showing good performance of 0.83 AUC. APD_50_ variability showed moderate outcomes for classifying intermediate-risk and low-risk drugs as both 0.70 AUCs. The variabilities of qNet, CaD_50_, APD_90_, and dVm/dt_Max_repol_ were low for classifying low-risk drugs; in particular, CaD_50_ variability was abysmal at 0.25 AUC.

**FIGURE 4:**
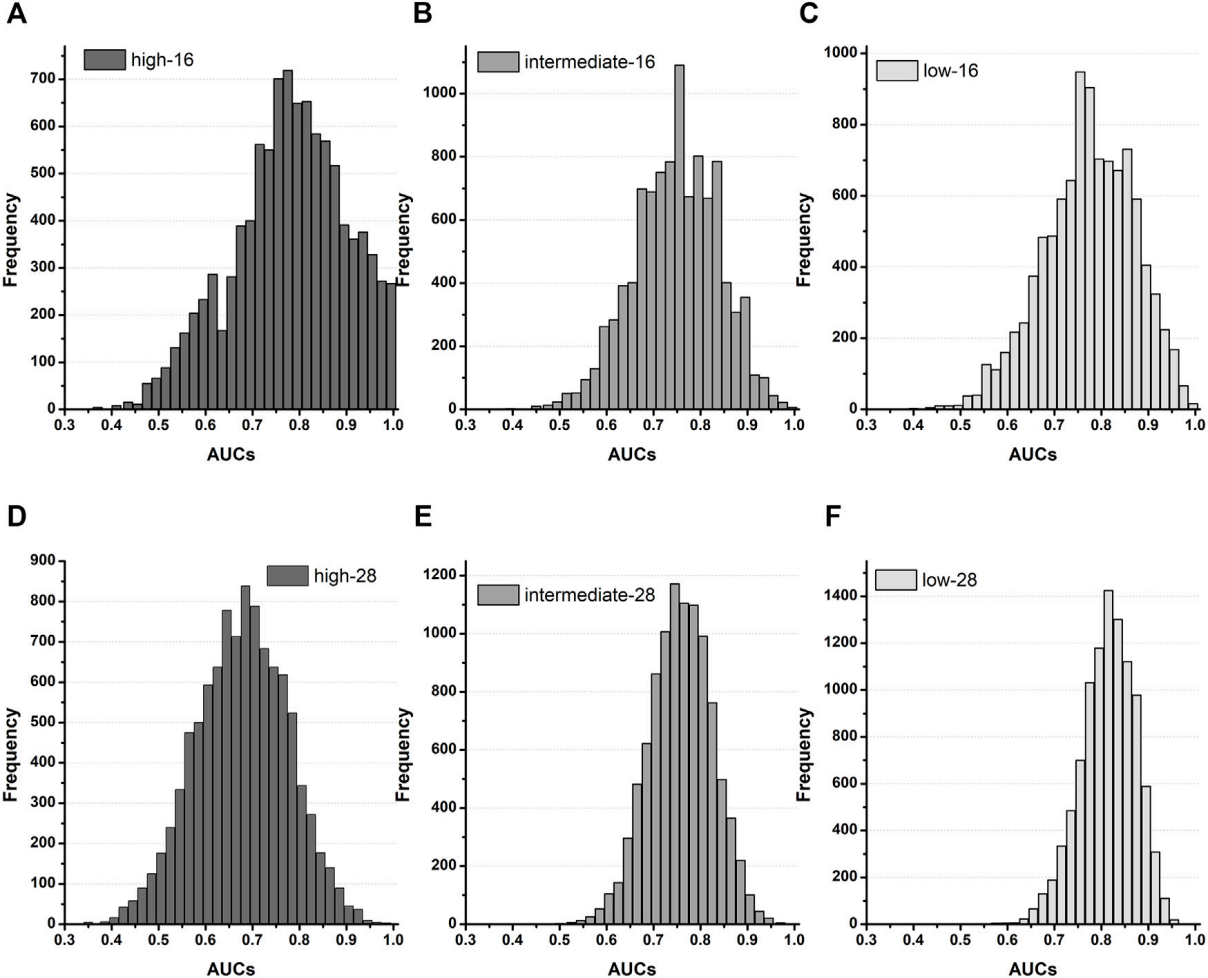
Distribution of AUCs based on the TdP-risk using qInward variability merged of three datasets; **(A–C)**, AUC distribution for the high, intermediate, and low-risk of the CNN classifier for 16 test drugs; **(D–F)**, AUC distribution for the high, intermediate, and low-risk for the CNN classifier for 28 drugs.

To validate the proposed CNN classifier, we compared the classification performance using the *in silico* feature variabilities computed from the three *in-vitro* datasets of Li dataset, Chantest dataset, and Nanion dataset (Tables 3, 4; Figure 4). Table 3 summarizes the proposed CNN classifiers for classifying 16 test drugs. When categorizing the high-risk and low-risk drugs in the combined datasets, the performances of the CNN classifier using qInward variability, which was the best model with an excellent level of AUC in Chantest datasets, decreased to moderate levels of 0.75 and 0.78 AUCs, respectively. Conversely, the classification performance of the intermediate-risk drugs increased a bit to 0.79 AUC when using qInward variability. The CNN classifier using dVm/dt_max_repol_ was the best when classifying the TdP-risk for 16 test drugs of combined datasets, but the performances were just medium levels as 0.77: high-risk, 0.79 for intermediate-risk, and 0.80 for low-risk (Table 3; Supplementary Figure S2). Two CNN classifiers using qInward variability and dVm/dt_max_repol_ variability satisfied the minimally acceptable LR+ and LR-levels in the three TdP-risks.

**TABLE 3:** CNN classifier performance for 16 test drugs according to the *in silico* feature variabilities of a merged dataset; performance indexes represent the median, the minimum, and the maximum values as the results of 10,000 times test algorithms; Three asterisks (***) denote excellent performance over 0.9 of the median AUC value, two asterisks (**) for good performance over 0.8 of the median AUC value, and one asterisk (*) for moderate performance over 0.7 of the median AUC value.

Model		In-silico feature variability of a combined dataset						
		qNet	qInward	CaD_50_	CaD_90_	APD_50_	APD_90_	dVm/dt_max_repol_
AUC	High	0.54 (0.10–0.72)	0.75* (0.35–1.00)	0.75* (0.15–1.00)	0.38 (0.11–0.94)	0.60 (0.02–1.00)	0.54 (0.06–1.00)	0.77* (0.10–1.00)
	Intermediate	0.71* (0.28–1.00)	0.79* (0.39–1.00)	0.71* (0.25–0.99)	0.67 (0.22–0.98)	0.76* (0.42–1.00)	0.79* (0.44–1.00)	0.79* (0.49–1.00)
	Low	0.75* (0.39–1.00)	0.78* (0.37–1.00)	0.55 (0.17–0.94)	0.65 (0.22–0.99)	0.64 (0.27–0.92)	0.65 (0.35–0.94)	0.80** (0.39–1.00)
LR+	High	5.00 (0.00–Inf)	5.33 (0.00–Inf)	5.0 (0.00–Inf)	5.0 (0.00–9.00)	5.0 (0.00–Inf)	5.0 (0.00–15.00)	5.0 (0.00–Inf)
	Intermediate	4.00 (0.8–Inf)	4.08 (0.83–Inf)	3.21 (0.41–Inf)	2.31 (0.51–Inf)	Inf (0.86–Inf)	Inf (0.86–Inf)	5.33 (0.85–Inf)
	Low	2.89 (0.48–5.67)	2.89 (0.37–8.0)	1.6 (0.00–9.6)	1.6 (0.00–7.5)	2.0 (0.00–5.67)	2.17 (0.00–5.67)	3.25 (0.92–15.0)
LR−	High	0.00 (0.00–1.55)	0.00 (0.00–1.45)	0.49 (0.00–2.77)	0.00 (0.00–1.45)	0.00 (0.00–0.15)	0.00 (0.00–1.55)	0.00 (0.00–1.50)
	Intermediate	0.48 (0.27–1.20)	0.46 (0.20–1.17)	0.49 (0.00–2.7)	0.62 (0.27–2.46)	0.46 (0.25–1.14)	0.44 (0.25–1.14)	0.42 (0.00–1.05)
	Low	0.43 (0.00–1.37)	0.43 (0.00–1.53)	0.8 (0.00–2.2)	0.80 (0.00–1.62)	0.67 (0.00–1.71)	0.65 (0.00–1.56)	0.42 (0.00–1.05)
Accuracy		0.59 (0.39–0.89)	0.66 (0.34–0.89)	0.59 (0.25–0.84)	0.57 (0.33–0.76)	0.64 (0.46–0.8)	0.64 (0.41–0.80)	0.67 (0.40–0.90)
F1 score		0.64 (0.48–0.84)	0.65 (0.32–0.88)	0.56 (0.24–0.82)	0.52 (0.29–0.74)	0.59 (0.38–0.78)	0.59 (0.35–0.78)	0.65 (0.38–0.89)

**TABLE 4:** CNN classifier performance for all 28 drugs according to the *in silico* feature variabilities of a merged dataset; performance indexes represent the median, the minimum, and the maximum values as the results of 10,000 times test algorithms; Three asterisks (***) denote excellent performance over 0.9 of the median AUC value, two asterisks (**) for good performance over 0.8 of the median AUC value, and one asterisk (*) for moderate performance over 0.7 of the median AUC value.

Model		In-silico feature variability of a combined dataset						
		qNet	qInward	CaD_50_	CaD_90_	APD_50_	APD_90_	dVm/dt_max_repol_
AUC	High	0.68 (0.21–0.99)	0.68 (0.38–0.94)	0.62 (0.33–0.98)	0.68 (0.17–0.92)	0.68 (0.17–0.99)	0.64 (0.16–0.98)	0.68 (0.18–0.99)
	Intermediate	0.75* (0.47–0.95)	0.75* (0.48–0.98)	0.74* (0.41–0.96)	0.71* (0.39–0.96)	0.67 (0.40–0.97)	0.81** (0.55–0.98)	0.81** (0.56–0.98)
	Low	0.74* (0.43–1.00)	0.82** (0.57–0.96)	0.68 (0.33–0.92)	0.54 (0.36–0.92)	0.76* (0.43–0.98)	0.74* (0.46–0.92)	0.70* (0 0.43–0.99)
LR+	High	4.0 (0.00–8.67)	3.25 (0.00–20.13)	2.89 (0.00–12.0)	3.86 (0.00–5.8)	2.62 (0.00–Inf)	4.0 (0.00–7.67)	3.0 (0.0–8.67)
	Intermediate	6.0 (1.14–Inf)	3.94 (0.93–Inf)	3.56 (0.56–Inf)	3.36 (0.86–Inf)	2.0 (0.42–Inf)	6.67 (1.2–Inf)	7.76 (1.4–Inf)
	Low	3.14 (0.82–8.0)	3.82 (1.11–10.5)	1.83 (0.00–10.67)	2.3 (0.48–8.8)	2.19 (0.46–7.33)	3.07 (0.75–8.33)	3.61 (0.78–17.78)
LR−	High	0.00 (0.00–1.53)	0.40 (0.00–1.53)	0.43 (0.00–1.60)	0.00 (0.00–1.58)	0.57 (0.00–1.6)	0.00 (0.0–1.58)	0.43 (0.0–1.53)
	Intermediate	0.5 (0.28–0.91)	0.49 (0.18–1.05)	0.51 (0.16–1.35)	0.58 (0.00–1.11)	0.65 (0.25–1.66)	0.47 (0.27–0.9)	0.45 (0.22–0.83)
	Low	0.35 (0.00–1.08)	0.40 (0.11–0.95)	0.7 (0.00–1.64)	0.58 (0.00–1.38)	0.63 (0.00–1.38)	0.46 (0.0–1.12)	0.35 (0.0–1.1)
Accuracy		0.63 (0.46–0.78)	0.64 (0.42–0.86)	0.58 (0.29–0.81)	0.58 (0.38–0.75)	0.53 (0.27–0.8)	0.64 (0.45–0.77)	0.65 (0.47–0.84)
F1 score		0.59 (0.41–0.77)	0.62 (0.38–0.86)	0.55 (0.30–0.81)	0.54 (0.38–0.75)	0.52 (0.27–0.79)	0.61 (0.41–0.77)	0.62 (0.43–0.83)

Table 4 summarizes the model performances in classifying TdP-risk for all 28 drugs using *in silico* feature variabilities of merged datasets. The proposed CNN classifier showed poor accuracy in classifying the high-risk drugs with AUC ranging from 0.62 to 0.68 in all the *in silico* feature variabilities. The CNN classifier using qInward variability categorized low-risk drugs to acceptable levels of 0.82 AUC and intermediate-risk drugs to moderate levels of 0.75 AUC. In the combined dataset, the classification performances for intermediate-risk drugs among all 28 drugs and 16 test drugs were modest in most of the *in silico* feature variabilities. However, when using APD_90_ variability and dVm/dt_Max_repol_ variability, the intermediate-risk drugs among all 28 drugs did not demonstrate high performance but were classified reasonably with an AUC of 0.81. For detecting the high-risk drugs, qNet variability showed good performance in 16 test drugs of Chantest dataset but poor performance in both 16 test drugs and all 28 drugs of merged datasets, which were 0.54 and 0.68 AUCs, respectively. Conversely, the classification performances for intermediate-risk and low-risk drugs using the CNN classifier using qNet variability increased to medium levels (approximately 0.75 for 16-test drugs and all 28 drugs in the combined datasets.

We compared the classification performance of the proposed CNN classifier using the variability of *in silico* biomarkers with that of the OLR classifier using a single value for *in silico* biomarkers (Supplementary Table S2). The single values of qNet, APD50, APD90, and dVm/dt_max_repol_ classified high-risk drugs with excellent accuracy and low-risk drugs with moderate accuracy for the 16 test drugs in the Chantest dataset. In the merged dataset, the high-risk drugs were moderately distinguished by qNet, dVm/dt_Max_repol_, APD50, and APD90, and the low-risk drugs could be roughly classified by only qNet and dVm/dt_Max_repol_, but not very accurately; their AUCs ranged from 0.7 to 0.79. However, the classification performance for intermediate-risk drugs was poor in all OLR classifiers using single values, less than 0.6 AUC. However, the CNN classifiers using the variabilities of qInward and dVm/dt_Max_repol_ could roughly classify the 16-test drugs into three levels of TdP risk, despite not being perfect.

## 4 Discussion

This study suggested a CNN classifier using the variability of *in silico* biomarkers to assess the TdP-risk of drugs under the hypothesis that the variability of *in silico* biomarkers is more likely to have more features based on the TdP-risk than every single *in silico* biomarker. Hinterseer et al. suggested that the variability of QT intervals based on beats affects drug-induced long-QT syndrome (Hinterseer et al., 2008). The mechanism of pharmaceutically occurring QT interval prolongation involves the blockage of ionic channels. Because *in silico* TdP metrics are obtained from AP and ionic currents computed from simulations with blockages of ionic channels as drug responses, the variability of the *in silico* TdP metric according to the beats also reflects the characteristics of torsadogenic drugs. Indeed, among the *in silico* features used in this study, the variabilities of qInward, CaD_50_, and CaD_90_ were better for classifying the TdP-risk of drugs, especially for intermediate-risk and low-risk drugs, than their single values. Notably, the CNN classifier using qInward variability was the best model to classify the high- and low-risk in the 16-test drugs of the Chantest datasets.

The single values of the *in silico* features were used to classify proarrhythmic risk using the threshold value derived from the OLR model (Li et al., 2019). The OLR model determined each threshold based on its distribution according to the main classification targets, such as high-risk, intermediate/low-risk, low-risk, and high/intermediate-risk. Therefore, the OLR model using the single values of the *in silico* features showed good performance for high-risk and low-risk drugs, but low performance for intermediate-risk drugs (Supplementary Table S2). Among the single *in silico* features, qNet, APD_50_, APD_90_, and dVm/dt_Max_repol_ were able to classify high-risk drugs well for 16-drugs in the Chantest datasets. The accuracies of APD_50_ and APD_90_ were higher than those of the CNN classifier using the qInward variability. However, the overall classification performances for the three proarrhythmic risks were better in the CNN classifier using qInward variability than in the OLR models. We believe this is because of the loss function used when training the CNN classifier, categorical cross-entropy, which updated the trainable parameters such as weights and bias to improve the classification accuracies of the three risk levels.

In this study, the CNN classifier using qInward variability exhibited the best performance in categorizing the proarrhythmic risk of drugs. In the 16 test drugs of Chantest data set, no other risk drugs were included among the drugs classified as high-risk through CNN classifiers using qInward variability (LR + > 10 for high-risk drugs). However, high-risk drugs can likely be classified into other risk groups (LR−= 0.27, high-risk drugs). On the other hand, for the 16 test drugs of the combined datasets, the possibility for high-risk drugs to be classified as other-risk was almost zero in the CNN classifier using qInward variability (LR-for high-risk drugs; a median of 0.00, and range of 0.00–1.45). The proposed model has little possibility of classifying low-risk drugs in the Chantest datasets as other-risk drugs, but the classified drugs as low-risk drugs may be other proarrhythmic risks (LR+ = 4.67 and LR- = 0.00 and low-risk drugs) (Roman et al., 2002).

We validated the classification performance of the proposed CNN model using *in silico* feature variabilities computed from three *in-vitro* datasets. Based on the results for the merged dataset, the proposed CNN classifier appeared to be specialized in classifying intermediate- and low-risk drugs. We believe that the machine learning features extracted from qInward variability through the proposed CNN classifier had the advantageous characteristics of determining intermediate- and low-risk drugs. However, the classification performance showed significant differences according to the used *in-vitro* datasets used. In addition, the proposed CNN classifier using qInward variability was under fitted to the 12-training datasets; thus, the classification performance for all 28-drugs was less than that for 16 test drugs, even in the Chantest dataset (Supplementary Table S3; Supplementary Figure S2). These results suggest the need for the proposed CNN model to be trained and tested using various types of drugs.

Because the *in silico* simulation results depend on *in-vitro* experimental data, many researchers have tried to validate the robustness of *in silico* features in classifying the proarrhythmic risk of drugs. Some researchers have validated the *in silico* features using uncertainty quantification algorithms and population modeling. Llopis-Lorente et al. improved the accuracy of the drug-induced TdP risk assessment by considering population variability (Llopis-Lorente et al., 2022). Chang et al. validated the robustness of TdP risk separation using qNet value and uncertainty quantification algorithms (Chang et al., 2017a). Han et al. suggested the selection method of calibration drugs and calibrated the previous OLR model by validating it using two lab-specific datasets (Han et al., 2020). In a previous study, we validated 12 promising *in silico* features using OLR, based on *in-vitro* datasets used in the AP simulation (Jeong et al., 2022a). In this study, we determined the CNN classifier using the variability of *in silico* features calculated using the Chantest dataset and validated the corresponding robustness using three merged *in-vitro* datasets.

This study has several limitations. First, we did not consider the mechano-electric feedback, which indicates that the mechanical contraction of the cell affects electrophysiological activity. Second, in an actual body environment, heart rate varies with time. Drugs can individually affect QT interval and heart rate, and the complexity of the QT interval assessment increases owing to heart rate variations (Roden, 2004). This study used the *in silico* feature variability under the static heart rate condition, which was 30 bpm, to mimic the bradycardia condition, without varying the heart rate in real time. We did not consider heart rate variability when performing *in silico* simulations. Therefore, the *in silico* simulation results could be slightly different from the clinical outcomes. However, as the tendency of the drug to affect the ionic channels is the same, we speculate that these limitations do not critically affect the results and do not change the entire conclusion of the study.

## Data Availability

The original contributions presented in the study are included in the article/Supplementary Materials, further inquiries can be directed to the corresponding author.

## References

[B1] Chang K. C. Dutta S. Mirams G. R. Beattie K. A. Sheng J. Tran P. N. (2017a). Uncertainty quantification reveals the importance of data variability and experimental design considerations for *in silico* proarrhythmia risk assessment. Front. Physiol. 8, 917–17. 10.3389/fphys.2017.00917 29209226 PMC5702340

[B2] Chang K. C. Dutta S. Mirams G. R. Beattie K. A. Sheng J. Tran P. N. (2017b). Uncertainty quantification reveals the importance of data variability and experimental design considerations for *in silico* proarrhythmia risk assessment. Front. Physiol. 8, 917–17. 10.3389/fphys.2017.00917 29209226 PMC5702340

[B3] Dutta S. Chang K. C. Beattie K. A. Sheng J. Tran P. N. Wu W. W. (2017). Optimization of an *in silico* cardiac cell model for proarrhythmia risk assessment. Front. Physiol. 8, 1–15. 10.3389/fphys.2017.00616 28878692 PMC5572155

[B4] Han X. Samieegohar M. Ridder B. J. Wu W. W. Randolph A. Tran P. (2020). A general procedure to select calibration drugs for lab-specific validation and calibration of proarrhythmia risk prediction models: An illustrative example using the CiPA model. J. Pharmacol. Toxicol. Methods 105, 106890. 10.1016/j.vascn.2020.106890 32574700

[B5] Hinterseer M. Thomsen M. B. Beckmann B. M. Pfeufer A. Schimpf R. Wichmann H. E. (2008). Beat-to-beat variability of QT intervals is increased in patients with drug-induced long-QT syndrome: A case control pilot study. Eur. Heart J. 29, 185–190. 10.1093/eurheartj/ehm586 18156612

[B6] International Council on Harmonisation (2005a). ICH E14 guideline: The clinical evaluation of QT/QTc interval prolongation and proarrhythmic potential for non-antiarrhythmic drugs E14. Fed. Regist. 70, 61134–61135. Available at: https://www.ich.org/page/efficacy-guidelines. 16237860

[B7] International Council on Harmonisation (2005b). ICH S7B guideline: The non-clinical evaluation of the potential for delayed ventricular repolarization (QT interval prolongation) by human pharmaceuticals S7B. Fed. Regist. 70, 61133–61134. https://www.ich.org/page/safety-guidelines. 16237859

[B8] Jeong D. U. Danadibrata R. Z. Marcellinus A. Lim K. M. (2022a). Validation of *in silico* biomarkers for drug screening through ordinal logistic regression. Front. Physiol. 13, 1–11. 10.3389/fphys.2022.1009647 PMC9583152 36277213

[B9] Jeong D. U. Yoo Y. Marcellinus A. Kim K. Lim K. M. (2022b). Proarrhythmic risk assessment of drugs by dVm/dt shapes using the convolutional neural network. CPT. Pharmacometrics Syst. Pharmacol. 11, 653–664. 10.1002/psp4.12803 35579100 PMC9124356

[B10] Lancaster M. C. Sobie E. A. (2016). Improved prediction of drug-induced Torsades de Pointes through simulations of dynamics and machine learning algorithms. Clin. Pharmacol. Ther. 4, 371–379. 10.1002/cpt.367 PMC6375298 26950176

[B11] Li Z. Dutta S. Sheng J. Tran P. N. Wu W. Chang K. (2017). Improving the *in silico* assessment of proarrhythmia risk by combining hERG (Human Ether-à-go-go-Related Gene) channel-drug binding kinetics and multichannel pharmacology. Circ. Arrhythm. Electrophysiol. 10, e004628–e004640. 10.1161/CIRCEP.116.004628 28202629

[B12] Li Z. Ridder B. J. Han X. Wu W. W. Sheng J. Tran P. N. (2019). Assessment of an in silico mechanistic model for proarrhythmia risk prediction under the CiPA initiative. Clin. Pharmacol. Ther. 105, 466–475. 10.1002/cpt.1184 30151907 PMC6492074

[B13] Llopis-Lorente J. Gomis-Tena J. Cano J. Romero L. Saiz J. Trenor B. (2020). *In silico* classifiers for the assessment of drug proarrhythmicity. J. Chem. Inf. Model. 60, 5172–5187. 10.1021/acs.jcim.0c00201 32786710

[B14] Llopis-Lorente J. Trenor B. Saiz J. (2022). Considering population variability of electrophysiological models improves the *in silico* assessment of drug-induced torsadogenic risk. Comput. Methods Programs Biomed. 221, 106934. 10.1016/j.cmpb.2022.106934 35687995

[B15] O’Hara T. Virág L. Varró A. Rudy Y. (2011). Simulation of the undiseased human cardiac ventricular action potential: Model formulation and experimental validation. PLoS Comput. Biol. 7, e1002061–e1002090. 10.1371/journal.pcbi.1002061 21637795 PMC3102752

[B16] Onakpoya I. J. Heneghan C. J. Aronson J. K. (2016). Post-marketing withdrawal of 462 medicinal products because of adverse drug reactions: A systematic review of the world literature. BMC Med. 14, 10–11. 10.1186/s12916-016-0553-2 26843061 PMC4740994

[B17] Roden D. M. (2004). Drug-induced prolongation of the QT interval. N. Engl. J. Med. 350, 1013–1022. 10.1056/NEJMra032426 14999113

[B18] Roman J. Gordon H., G. David L., S. (2002). III. How to use an article about a diagnostic test. A. What are the results and will they help me in caring for my patients? Evid. Based. Nurs. 5, 8. 10.1136/ebn.5.1.8

[B19] Sager P. T. Gintant G. Turner J. R. Pettit S. Stockbridge N. (2014). Rechanneling the cardiac proarrhythmia safety paradigm: A meeting report from the cardiac safety research consortium. Am. Heart J. 167, 292–300. 10.1016/j.ahj.2013.11.004 24576511

[B20] Strauss D. G. Gintant G. Li Z. Wu W. Blinova K. Vicente J. (2019). Comprehensive *in vitro* proarrhythmia assay (CiPA) update from a cardiac safety research consortium/ health and environmental Sciences Institute/ FDA meeting. Ther. Innov. Regul. Sci. 53, 519–525. 10.1177/2168479018795117 30157676

[B21] Tomek J. Bueno-Orovio A. Passini E. Zhou X. Minchole A. Britton O. (2019). Development, calibration, and validation of a novel human ventricular myocyte model in health, disease, and drug block. Elife 8, e48890–e48938. 10.7554/eLife.48890 31868580 PMC6970534

[B22] Vicente J. Zusterzeel R. Johannesen L. Mason J. Sager P. Patel V. (2018). Mechanistic model-informed proarrhythmic risk assessment of drugs: Review of the “CiPA” initiative and design of a prospective clinical validation study. Clin. Pharmacol. Ther. 103, 54–66. 10.1002/cpt.896 28986934 PMC5765372

