# Supplementary material for: qInward variability-based in-silico proarrhythmic risk assessment of drugs using deep learning model
Source: Front Physiol. 2022 Dec 14;13:1080190. doi: 10.3389/fphys.2022.1080190 (PMC9794579; doi:10.3389/fphys.2022.1080190)
Supplement: Supplementary file 1 [file DataSheet1.PDF]

Supplementary Material

qInward Variability-based In-silico Proarrhythmic Risk Assessment of Drugs using Deep Learning Model

Da Un Jeong[1], Nurul Qashri Mahardika T[1], Aroli Marcellinus[1], Ki Moo Lim[1, 2*]

*** Correspondence:** Ki Moo Lim, kmlim@kumoh.ac.kr

1 Supplementary Figures

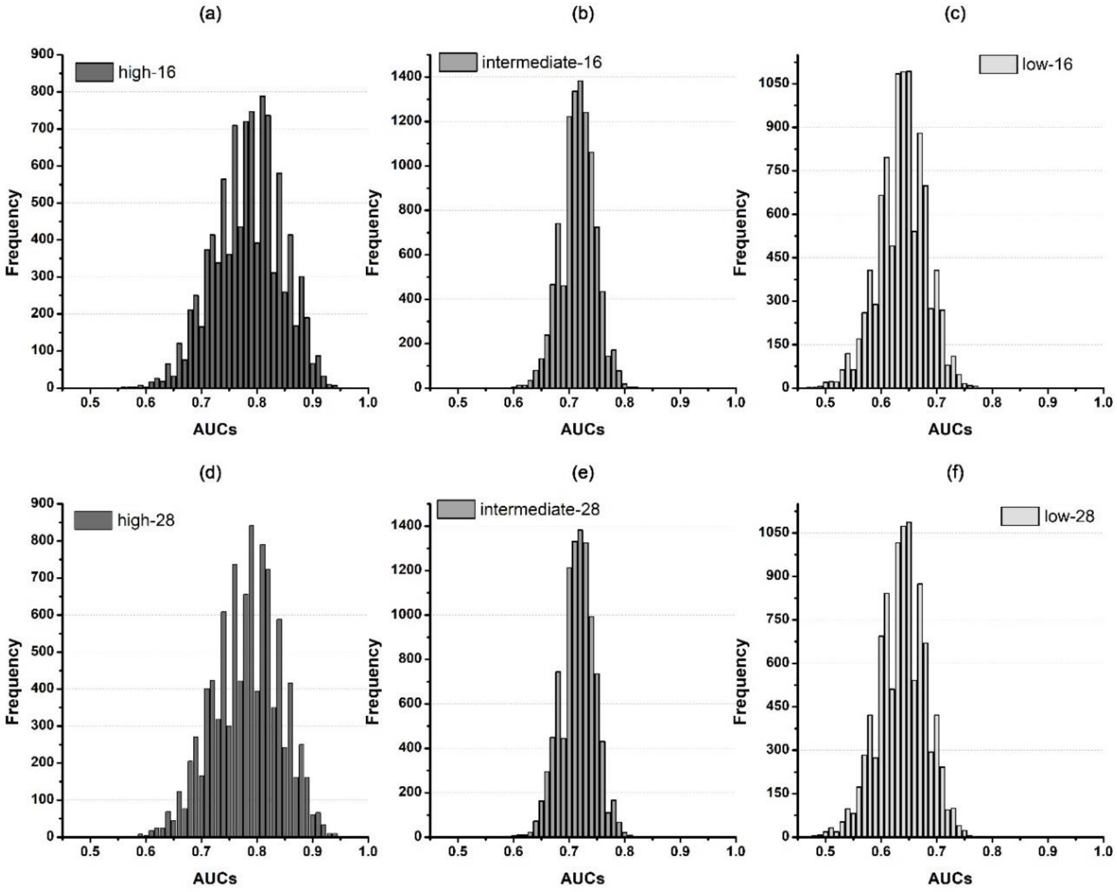

Supplementary Figure S 1. Distribution of AUCs based on the TdP-risk using dVm/dt$_{Max_repol}$ of Chantest et al.; (a, b, c), AUC distribution for the high, intermediate, and low-risk of the CNN classifier for 16 test drugs; (d, e, f), AUC distribution for the high, intermediate, and low-risk for the CNN classifier for all 28 drugs

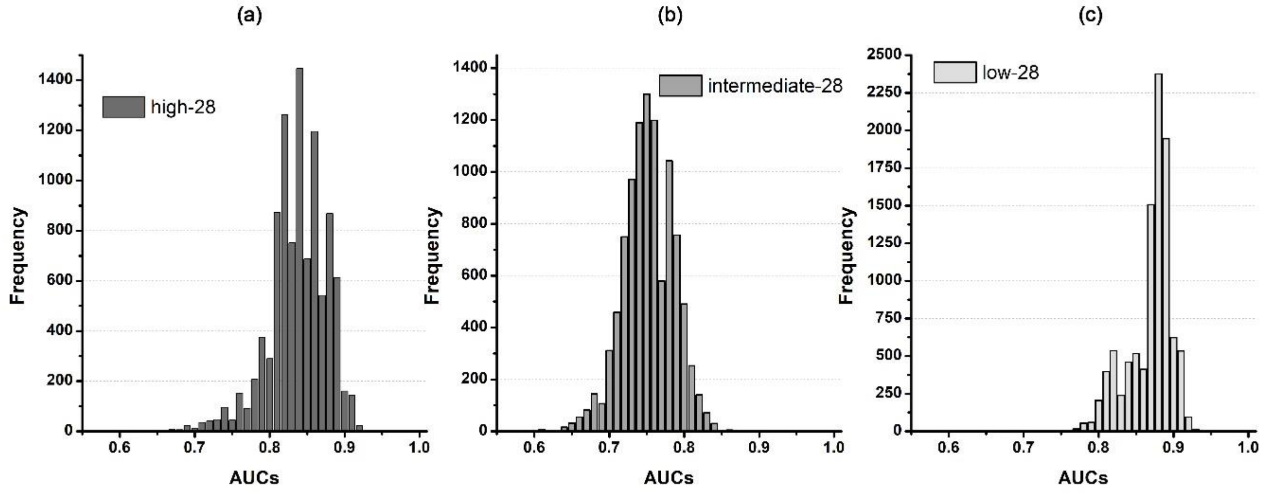

Supplementary Figure S 2. Distribution of AUCs based on the TdP-risk using qInward variability of all 28 drugs in the Chantest et al. study; (a, b, c) AUC distribution for the high, intermediate, and low risk of the CNN classifier for 16 test drugs

2 Supplementary Tables

Supplementary Table S 1 CNN classifier performance for 16 drugs of three datasets according to in silico feature variability; performance indexes represent the median, the minimum, and the maximum values as the results of 10,000 times test algorithms; Three asterisks (***) denote excellent performance over 0.9 of the median AUC value, two asterisks (**) for good performance over 0.8 of the median AUC value, and one asterisk (*) for moderate performance over 0.7 of the median AUC value.

Model		In-silico feature variability of the Chantest dataset						
		qNet	qInward	CaD_{50}	CaD_{90}	APD_{50}	APD_{90}	dVm/dt_{max_repol}
Li datasets	High	0.81** (0.35–1.0)	0.69 (0.25–0.99)	0.55 (0–0.98)	0.78* (0.32–1.0)	0.42 (0.24-0.88)	0.52 (0.25-0.92)	0.62 (0.25-0.62)
	Intermediate	0.68 (0.29–0.96	0.80** (0.47–1.00)	0.50 (0.14–0.86)	0.61 (0.18–1.0)	0.56 (0.33-0.75)	0.56 (0.33-0.79)	0.61 (0.44-0.79)
	Low	0.75* (0.35–1.0)	0.87** (0.31–1.00)	0.46 (0.02–1.0)	0.46 (0–0.94)	0.29 (0.09-0.73)	0.47 (0.11-0.84)	0.53 (0.27-0.80)
Chantest dataset	High	0.83** (0.31–0.96)	0.94*** (0.60–1.00)	0.83** (0.54–1.00)	0.46 (0.19–0.90)	0.82** (0.63–0.94)	0.81** (0.67–0.92)	0.81** (0.56–0.94)
	Intermediate	0.62 (0.38–0.78)	0.75* (0.57–0.92)	0.71* (0.48–0.84)	0.71* (0.57–0.86)	0.70* (0.55–0.80)	0.73* (0.57–0.84)	0.72* (0.60–0.82)
	Low	0.64 (0.44–0.98)	0.93*** (0.82–1.00)	0.25 (0.01–0.51)	0.71* (0.23–0.65)	0.70* (0.49–0.88)	0.51 (0.36–0.89)	0.65 (0.46–0.78)
Nanion datasets	High	0.83** (0.38-0.98)	0.21 (0.13-0.31)	0.34 (0.06-0.90)	0.62 (0.33-0.78)	0.52 (0.02-0.90)	0.23 (0.13-0.31)	0.19 (0.08-0.31)
	Intermediate	0.76* (0.42-0.97)	0.81** (0.71-0.9)	0.73* (0.41-0.92)	0.59 (0.24-0.84)	0.22 (0.00-0.43)	0.83** (0.63-0.98)	0.83** (0.71-0.92)
	Low	0.62 (0.45-0.87)	0.58 (0.51-0.71)	0.55 (0.32-0.77)	0.70* (0.36-0.82)	0.35 (0.16-0.62)	0.62 (0.44-0.75)	0.64 (0.53-0.75)

Supplementary Table S 2. Comparison of performances in the model using the variabilities of in-silico biomarkers and the model using a single value of in-silico biomarkers; performance indexes represent the median, the minimum, and the maximum values as the results of 1 0,000 times test algorithms; Three asterisks (***) denote excellent performance over 0.9 of the median AUC value, two asterisks (**) for go od performance over 0.8 of the median AUC value, and one asterisk (*) for moderate performance over 0.7 of the median AUC value.

	16-test drugs of the Chantest dataset			16-test drugs of merged datasets			28 drugs of merged datasets		
	High	Intermediate	Low	High	Intermediate	Low	High	Intermediate	Low
qNet	0.92***<br>(0.71 – 0.96)	0.52<br>(0.39 – 0.79)	0.76*<br>(0.67 – 0.91)	0.75*<br>(0.58 - 0.92)	0.59<br>(0.33 - 0.80)	0.76*<br>(0.57 - 0.85)	0.78*<br>(0.60 - 0.88)	0.58<br>(0.40 - 0.74)	0.73*<br>(0.62 - 0.84)
qNet variability	0.83**<br>(0.31 – 0.96)	0.62<br>(0.38 – 0.78)	0.64<br>(0.44 – 0.98)	0.54<br>(0.10 – 0.72)	0.71*<br>(0.28 – 1.00)	0.75*<br>(0.39 – 1.00)	0.68<br>(0.21 – 0.99)	0.75*<br>(0.47 – 0.95)	0.74*<br>(0.43 – 1.00)
qInward	0.62<br>0.46 – 0.71)	0.44<br>(0.33 – 0.64)	0.70*<br>(0.50 – 0.70)	0.62<br>(0.58 - 0.71)	0.57<br>(0.44 - 0.71)	0.70*<br>(0.55 - 0.70)	0.59<br>(0.53 - 0.66)	0.53<br>(0.41 - 0.64)	0.75*<br>(0.67 - 0.83)
qInward variability	0.94***<br>(0.60 – 1.00)	0.75*<br>(0.57 – 0.92)	0.93***<br>(0.82 – 1.00)	0.75*<br>(0.35 – 1.00)	0.79*<br>(0.39 – 1.00)	0.78*<br>(0.37 – 1.00)	0.68<br>(0.38 – 0.94)	0.75*<br>(0.48 – 0.98)	0.82**<br>(0.57 – 0.96)
CaD$_{50}$	0.12<br>(0.04 – 0.33)	0.50<br>(0.39 – 0.64)	0.37<br>(0.18 – 0.52)	0.21<br>(0.12 - 0.46)	0.57<br>(0.39 - 0.93)	0.43<br>(0.24 - 0.62)	0.28<br>(0.20 - 0.47)	0.55<br>(0.41 - 0.77)	0.46<br>(0.30 - 0.62)
CaD$_{50}$ variability	0.83**<br>(0.54 – 1.00)	0.71*<br>(0.48 – 0.84)	0.25<br>(0.01 – 0.51)	0.75*<br>(0.15 – 1.00)	0.71*<br>(0.25 – 0.99)	0.55<br>(0.17 – 0.94)	0.62<br>(0.33 – 0.98)	0.74*<br>(0.41 – 0.96)	0.68<br>(0.33 – 0.92)
CaD$_{90}$	0.12<br>(0.04 – 0.33)	0.50<br>(0.39 – 0.64)	0.37<br>(0.18 – 0.52)	0.25<br>(0.17 - 0.54)	0.52<br>(0.33 - 0.73)	0.43<br>(0.34 - 0.57)	0.33<br>(0.25 - 0.50)	0.52<br>(0.35 - 0.74)	0.49<br>(0.38 - 0.62)
CaD$_{90}$ variability	0.46<br>(0.19 – 0.90)	0.71*<br>(0.57 – 0.86)	0.71*<br>(0.23 – 0.65)	0.38<br>(0.11 – 0.94)	0.67<br>(0.22 – 0.98)	0.65<br>(0.22 – 0.99)	0.68<br>(0.17 – 0.92)	0.71*<br>(0.39 – 0.96)	0.54<br>(0.36 – 0.92)
APD$_{50}$	0.96***<br>(0.79 – 0.96)	0.57<br>(0.39 – 0.64)	0.77*<br>(0.63 – 0.82)	0.75*<br>(0.62 - 0.88)	0.53<br>(0.33 - 0.79)	0.67<br>(0.54 - 0.86)	0.75*<br>(0.60 - 0.86)	0.53<br>(0.32 - 0.70)	0.68<br>(0.54 - 0.81)
APD$_{50}$ variability	0.82**<br>(0.63 – 0.94)	0.70*<br>(0.55 – 0.80)	0.70*<br>(0.49 – 0.88)	0.60<br>(0.02 – 1.00)	0.76 *<br>(0.42 – 1.00)	0.64<br>(0.27 – 0.92)	0.68<br>(0.17 – 0.99)	0.67<br>(0.40 – 0.97)	0.76*<br>(0.43 – 0.98)
APD$_{90}$	0.96***<br>(0.79 – 0.96)	0.57<br>(0.39 – 0.64)	0.77*<br>(0.63 – 0.82)	0.75*<br>(0.67 - 0.88)	0.53<br>(0.33 - 0.79)	0.67<br>(0.49 - 0.86)	0.75*<br>(0.60 - 0.86)	0.53<br>(0.35 - 0.70)	0.65<br>(0.51 - 0.79)
APD$_{90}$ variability	0.81**<br>(0.67 – 0.92)	0.73*<br>(0.57 – 0.84)	0.51<br>(0.36 – 0.89)	0.54<br>(0.06 – 1.00)	0.79*<br>(0.44 – 1.00)	0.65<br>(0.35 – 0.94)	0.64<br>(0.16 – 0.98)	0.81**<br>(0.55 – 0.98)	0.74*<br>(0.46 – 0.92)
dVm/dt$_{Max_repol}$	0.92***<br>(0.75 – 0.96)	0.52<br>(0.39 – 0.66)	0.72*<br>(0.63 – 0.86)	0.79*<br>(0.58 - 1.00)	0.53<br>(0.33 - 0.80)	0.72*<br>(0.58 - 0.86)	0.76*<br>(0.58 - 0.88)	0.55<br>(0.38 - 0.73)	0.70*<br>(0.59 - 0.81)
dVm/dt$_{Max_repol}$ variability	0.81**<br>(0.56 – 0.94)	0.72*<br>(0.60 – 0.82)	0.65<br>(0.46 – 0.78)	0.77*<br>(0.10 – 1.00)	0.79*<br>(0.49 – 1.00)	0.80**<br>(0.39 – 1.00)	0.68<br>(0.18 – 0.99)	0.81**<br>(0.56 – 0.98)	0.70*<br>(0 .43 – 0.99)

Supplementary Table S 3. CNN classifier performance for all 28 drugs according to in silico feature variability; performance indexes r epresent the median, the minimum, and the maximum values as the results of 10,000 times test algorithms; Three asterisks (***) denote exce llent performance over 0.9 of the median AUC value, two asterisks (**) for good performance over 0.8 of the median AUC value, and one as terisk (*) for moderate performance over 0.7 of the median AUC value.

Model		In-silico feature variability of Chantest dataset						
		qNet	qInward	CaD_{50}	CaD_{90}	APD_{50}	APD_{90}	dVm/dt_{max_repol}
AUC	High	0.86** (0.51 – 0.93)	0.84** (0.64 – 0.94)	0.83** (0.43 – 0.95)	0.58 (0.28 – 0.78)	0.82* (0.61 – 0.94)	0.79* (0.61 – 0.94)	0.79* (0.57 – 0.95)
	Intermediate	0.68 (0.55 – 0.79)	0.75* (0.60 – 0.86)	0.62 (0.55 – 0.80)	0.72* (0.55 – 0.85)	0.70* (0.57 – 0.79)	0.71* (0.57 – 0.81)	0.72* (0.58 – 0.82)
	Low	0.77* (0.54 – 0.94)	0.88** (0.73 – 0.94)	0.64 (0.30 – 0.65)	0.52 (0.42 – 0.74)	0.70* (0.50 – 0.88)	0.70* (0.50 – 0.89)	0.65 (0.47 – 0.80)
LR+	High	4.00 (0.83 – Inf)	4.17 (1.22 – 9.00)	1.22 (0.00 – 4.60)	2.78 (0.00 – 6.00)	3.00 (0.83 – 14.78)	2.76 (0.7 – 14.78)	3.00 (0.70 – Inf)
	Intermediate	2.02 (0.96 – 8.67)	2.67 (0.97 – 10.0)	2.31 (0.96 – 4.5)	2.91 (1.04 – 10.00)	2.33 (0.96 – 4.12)	2.33 (1.04 – 4.50)	2.7 (1.04 – 10.00)
	Low	2.30 (0.71 – 6.00)	3.68 (1.71 – 6.00)	1.06 (0.60 – 2.00)	1.69 (0.92 – 4.67)	1.69 (0.00 – 3.75)	1.83 (0.38 – 3.75)	2.93 (0.75 – 4.8)
LR-	High	0.53 (0.16 – 1.07)	0.33 (0.16 – 0.92)	0.92 (0.33 – 1.03)	0.44 (0.00 – 1.50)	0.53 (0.00 – 1.07)	0.60 (0.00 – 1.14)	0.60 (0.00 – 1.14)
	Intermediate	0.63 (0.24 – 1.03)	0.58 (0.31 – 1.02)	0.61 (0.33 – 1.03)	0.55 (0.31 – 0.97)	0.56 (0.33 – 1.03)	0.56 (0.33 – 0.97)	0.52 (0.29 – 0.97)
	Low	0.54 (0.00 – 1.15)	0.35 (0.17 – 0.71)	0.97 (0.67 – 1.23)	0.75 (0.48 – 1.04)	0.75 (0.35 – 1.64)	0.69 (0.35 – 1.38)	0.43 (0.00 – 1.12)
Accuracy		0.57 (0.39 – 0.73)	0.61 (0.44 – 0.79)	0.46 (0.30 – 0.58)	0.55 (0.40 – 0.69)	0.54 (0.33 – 0.72)	0.54 (0.36 – 0.68)	0.58 (0.36 – 0.77)
F1 score		0.57 (0.39 – 0.71)	0.61 (0.43 – 0.79)	0.43 (0.29 – 0.57)	0.50 (0.36 – 0.68)	0.54 (0.32 – 0.71)	0.54 (0.36 – 0.68)	0.57 (0.36 – 0.95)
